# Multidrug-Resistant *Pseudomonas aeruginosa* Evokes Differential Inflammatory Responses in Human Microglial and Retinal Pigment Epithelial Cells

**DOI:** 10.3390/microorganisms8050735

**Published:** 2020-05-14

**Authors:** Poonam Naik, Sukhvinder Singh, Sushma Vishwakarma, Inderjeet Kaur, Vivek Pravin Dave, Ashok Kumar, Joveeta Joseph

**Affiliations:** 1 Jhaveri Microbiology Centre, Brien Holden Eye Research Centre, L. V. Prasad Eye Institute, Hyderabad, Telangana 500034, India; naikpoonam92@gmail.com; 2 Manipal Academy of Higher Education, Manipal, Karnataka 576104, India; svishwakarma17@gmail.com; 3 Department of Ophthalmology, Visual and Anatomical Sciences, Wayne State University, Detroit, Michigan, MI 48201, USA; gp0153@wayne.edu (S.S.); akuma@med.wayne.edu (A.K.); 4 Kallam Anji Reddy Molecular Genetics laboratory, Jhaveri Microbiology Centre, Brien Holden Eye Research Centre, L. V. Prasad Eye Institute, Hyderabad, Telangana 500034, India; inderjeet@lvpei.org; 5 Smt. Kannuri Santhamma Centre for vitreoretinal diseases. L V Prasad Eye Institute, Hyderabad, Telangana 500034, India; vivekdave@lvpei.org

**Keywords:** microglia, RPE, *P. aeruginosa*, endophthalmitis, innate immune response

## Abstract

Increasing incidences of multidrug-resistant (MDR) pathogens causing endophthalmitis threaten our ability to treat this condition, and the modulation of inflammatory responses by MDR bacteria is not known. In this study, using human microglia and retinal pigment epithelial (RPE) cells, we compare the inflammatory responses of sensitive (S-*PA*) and multidrug-resistant (MDR-*PA*) clinical isolates of *Pseudomonas aeruginosa*. Infected cells were subjected to qPCR analysis, enzyme-linked immunosorbent assay (ELISA), and immunostaining to assess the expression of inflammatory mediators. Both microglia and RPE cells, challenged with S-*PA* and MDR-*PA*, induced a time-dependent expression of inflammatory cytokines. Significant differences were observed in expression levels of Toll-like receptors (TLR) TLR4, TLR5, and TLR9 in microglia cells challenged with MDR-*PA* vs. S-*PA*. Similarly, mRNA levels of interleukin (IL)-6, tumor necrosis factor (TNF)-α, Interferon (IFN)-γ, and matrix metalloproteinase (MMP)-9 were also higher in MDR-*PA*-infected cells. At protein levels, upregulation was observed for IL-10 (*p* = 0.004), IL-8 (*p* = 0.0006), IL-1β (*p* = 0.02), and Granulocyte-macrophage colony-stimulating factor (GM-CSF) (*p* = 0.0006) in cells infected MDR-*PA* versus S-*PA* in both microglia and RPE cells; however, the response was delayed in RPE cells. Heatmap and STRING analysis highlighted the existence of a cross-talk between the inflammatory and cytokine-mediated signaling pathways. Our study highlights a differential inflammatory response evoked by MDR vs. sensitive pathogens in retinal cells during endophthalmitis.

## 1. Introduction

Multidrug-resistant (MDR) bacterial endophthalmitis remains a highly fulminant inflammatory condition, with a worse visual prognosis than infection by sensitive bacterial pathogens [1]. By the competitive persistence of resistant organisms in the environment, these pathogens propagate and cause significant medical co-morbidities. The clinical manifestation of these infections reflects a complex interplay between the host tissues and pathogen and minimum inhibitory concentration of the antibiotics. There is evidence in vivo that antibiotics work in conjunction with host immunity, and that a strong immune defense can significantly bring down the duration of treatments, as has been reported with some acute infections that have led to shorter drug treatments in hosts with robust immunity [2]. As the innate immune system is known to play a major role in host defense against bacterial infection, harnessing host’s innate responses together with appropriate antimicrobial treatment may reduce development of resistance, leading to successful therapy and the resolution of antimicrobial-resistant infections [3]. Despite recognizing the importance of the role of the host’s immune defenses, most studies focus only on the pharmacokinetics (PK) of the pathogen and the particular antibiotic, while the role of host immune response remains inconclusive [4,5,6,7]. Recent studies have shown that the immune response against antibiotic, MDR, and susceptible bacterial strains of the same species also may be significantly different [8,9]. Therefore, this inevitably raises the question about the role of immune mediators, such as pro- and anti-inflammatory cytokines, along with Toll-like receptors (TLRs), during MDR bacterial infections.

Because MDR pathogens are emerging as a particularly serious challenge in the management of ocular infections in the present study, we focused on MDR bacteria causing endophthalmitis. Our study in India has reported that multidrug resistance was more common in Gram-negative than Gram-positive bacteria (78.6% vs. 21.4%), and MDR *Pseudomonas aeruginosa* was found to be the most common cause of endophthalmitis. Additionally, we found a rapid progression of infection, resulting in poor visual outcome despite prompt antibiotic treatment in nearly 71% of the patients with MDR endophthalmitis [1]. It has been previously shown that innate responses in the eye’s retinal pigment epithelial (RPE) cells may serve as the crucial role in the defense against invading pathogens in the retina [10]. Additionally, the microglia, representing the resident macrophages of the central nervous system (CNS) [11], and the retina are also involved in antimicrobial defense [12], and are uniquely poised to evoke innate responses against foreign pathogens, even prior to leukocyte infiltration. Their ability to respond to pathogens us due to the expression of a vast repertoire of innate signals, which include the Toll-like receptors (TLRs) and pattern recognition receptors, which together function to detect and eliminate invading microbes [13,14]. Hence, we hypothesized that RPE and microglial cells might exert a differential immune response to a clinical strain of MDR *P. aeruginosa* (MDR-*PA*) compared to a sensitive strain of *P. aeruginosa* (S-*PA*) by modulating the anti-inflammatory activities and simultaneously affecting innate host immunity.

## 2. Materials and Methods

### 2.1. Ethics Statement

All procedures were approved by the Institutional Review Board of the L V Prasad Eye Institute (LEC 09-18-125) dated 11 September 2018.

### 2.2. Characterization of Pseudomonas aeruginosa Strains

Strains used in the study included clinical strains isolated from the vitreous of patients diagnosed clinically with infectious endophthalmitis after routine microbiological work-up and antibiotic susceptibility testing. For antibiotic susceptibility testing, a minimum inhibitory concentration (MIC) was determined using Ezy MIC strips (Himedia Laboratories, Telangana, India) or VITEK 2 AST cards, according to manufacturer’s protocol and following Clinical and Laboratory Standards Institute (CLSI) guidelines [15]. The isolates were screened for susceptibility towards chloramphenicol, fluoroquinolones (such as ciprofloxacin, moxifloxacin, gatifloxacin, ofloxacin, and levofloxacin), aminoglycosides (such as gentamycin, amikacin, and tobramycin), polymyxins (like colistin), cephalosporins (like ceftazidime and cefepime), carbepenems (such as imipenem, doripenem, and meropenem), glycycline (such as tigercycline and ureidopenicillins), and β-lactam inhibitors (such as piperacillin/tazobactum, ticarcillin/clavulanic acid, and cefoperazone/subalactam). Multi-drug resistance was defined as acquired non-susceptibility to at least one agent in three or more antimicrobial categories.

The susceptible strain S*-PA* (L-2050/18) was susceptible to all routine antibiotics tested, and the resistant strain, *MDR-PA* (L-2051/18), was multi-drug/pan-resistant to all antibiotics. The details are provided in Supplementary Table S1. Bacteria were plated on Mueller Hinton agar (MHA) (Himedia, India) from a frozen stock. Following 24 h incubation, 1–2 colonies were transferred to 1 mL of Brain Heart Infusion broth to achieve 0.5 McFarland (1 × 10^8^ cells/mL). The broth culture was incubated overnight (12–16 h) at 37 °C with shaking, and the cell viability was evaluated by colony-forming unit (CFU) assay in each preparation.

### 2.3. RPE and Microglia Cell Culture, Infection with P. aeruginosa Strains and Quantification

The immortalized human microglial cell line (CHME-3) and retinal pigment epithelial cell line (ARPE-19) used in the study were maintained in DMEM-F12, supplemented with fetal bovine serum and antibiotic cocktail at 37 °C in a 5% CO_2_ humidified incubator. Prior to setting up the experiments, the cells were grown in serum and antibiotic-free medium prior to infection, and seeded into a 6-well plate. The monolayer formed was co-cultured with clinical strains of *Pseudomonas aeruginosa* (MDR-*PA* + S-*PA*) with a multiplicity of infection of 10:1 for up to 24 h; uninoculated wells served as uninfected controls. The same passage number of both cell lines was maintained for the study of responses to both MDR-*PA* + S-*PA* strains. Following 0, 1, 2, 4, 12, and 24 h of incubation at 37 °C, 5% CO_2_, the cells along with medium was removed and homogenized in PBS, and the number of bacteria in the lysate was assessed by viable counts after incubation at 37 °C for 24 h. Results were expressed as mean ± SD number of colony-forming units. The remaining lysate was clarified by centrifugation and stored at −80 °C for further analysis.

### 2.4. Total RNA Extraction, cDNA Synthesis and Real-Time Reverse Transcriptase-Polymerase Chain Reaction (RT-PCR) Analysis

The total RNA was extracted according to the manufacturer’s instructions from lysates of all time points, using the RNeasy mini kit (Qiagen Ltd., West Sussex, United Kingdom). The RNA was purified and quantified using the Nano-Drop spectrophotometer (Nanovue plus). First-strand cDNA was synthesized from equal RNA amounts (2 μg) using the Verso cDNA Synthesis Kit (Thermo scientific). cDNA was further amplified using primers for human Toll-like receptors 1–7 (TLR1–7; TLR8 and TLR10), interleukin (IL)-1α, IL-1β, tumor necrosis factor (TNF)-α, granulocyte-macrophage colony-stimulating factor (GM-CSF), IL-6, IL-8, IL-10, matrix metalloproteinase (MMP)-2 and -9, tissue inhibitor of metalloproteinases (TIMP-1), and β-actin (internal control), as described previously [16,17], using SYBR Green Master Mix (Thermo scientific) in a Step-One Plus real-time PCR system (Applied Biosystems, Foster City, CA, United States), with the following amplification conditions: initial denaturation of 10 min at 95 °C, followed by 40 cycles of 50° for 2 min, 95 °C for 10 min, 95 °C for 15 s, and 60 °C for 1 min. To confirm the specificity of the reaction, melting curve analysis was carried out. Further, the Ct values for each gene amplification were normalized with respect to the house-keeping gene, β-actin, by the 2^−ΔΔCT^ method, and the expression levels are presented as fold induction compared to uninfected cells. The RT-PCR products, along with the internal control β-actin, were also subjected to electrophoresis on 2% EtBr agarose gels.

### 2.5. Cytokine Estimation by ELISA (Enzyme-Linked Immunosorbent Assay)

For the quantification of cytokine secretion, 100 µl of supernatants recovered post-infection were assayed for secreted GM-CSF, IL-10, IL-6, and IL-8 using multiplex, enzyme-linked immunosorbent assay (ELISA) kits from Millipore, using a MAGPIX multiplex analyzer. Values were normalized based on the total protein amount in the samples. The cytokine and chemokine concentrations were interpolated from standard curves. Values are expressed as the means from three separate experiments ± SEM for *n* ≥ 3 analyses per time point. Each sample was assayed in duplicate. The reported sensitivities of these assays are <0.35 pg/mL for GM-CSF, <0.56 pg/mL for IL-10, <0.11 pg/mL for IL-6, and <0.13 pg/mL for IL-8. MMP-9 and MMP-2 levels in culture supernatants were also measured using separate quantitative immunoassay ELISA kits (Sigma Aldrich, Merk, Bangalore, Karanataka). The absorbance was read in a BT 2000 Microkinetics Reader (Bio-Tek Instrument Inc, Mumbai, Maharashtra.) at 450 nm, and a standard curve was generated to quantify the MMPs. The minimum detectable dose of Human MMP-2 was determined to be 3500 pg/mL, and MMP-9 was determined to be 10 pg/mL.

### 2.6. Immunofluorescence Staining

CHME-3 cells were cultured on 12-well glass chamber slides and challenged with *P. aeruginosa* (MDR-*PA* + S-*PA*) for the above-mentioned time points. The cells were washed thrice with PBS and fixed in 4% paraformaldehyde. The cells were then permeabilized for 10 min (only for TLR4) with Triton X-100 at −20 °C. The fixed cells were then blocked in 2.5% (*w*/*v*) BSA for 1 h at room temperature, followed by incubation with primary antibodies (1:100 dilution) (rabbit monoclonal antibodies, Abcam) overnight at 4 °C. Following extensive washing with PBS, the cells were incubated for 1 h with fluorescein-conjugated secondary antibodies (1:200 dilution). Finally, the cells were again extensively washed with buffer, and the slides were mounted in DAPI and visualized using a fluorescence microscope (Carl Ziess AXIO 503 monoscope A1). The level of cellular fluorescence from images was quantified using Image J software. The uninfected cells were taken as control. The corrected total cell fluorescence was calculated from the three different individually captured images, and the mean values were plotted.

### 2.7. Statistical Analysis

GraphPad Prism (Version 5) was used for statistical analysis. Differences in the means were calculated using the two-tailed unpaired *t*-test or one-way analysis of variance (ANOVA): n.s., not significant (*p* > 0.05); *, *p* ≤ 0.05; **, *p* ≤ 0.01; and ***, *p* ≤ 0.001.

## 3. Results

### 3.1. Multidrug Resistant P. aeruginosa Are More Viable than Sensitive Strains in Infected Microglia and RPE Cells

**Figure 1:**
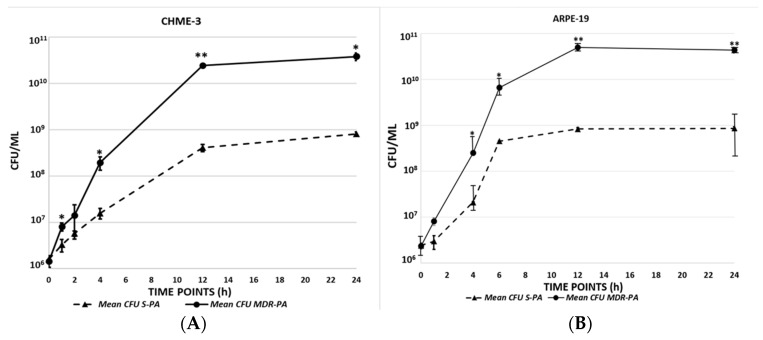
Human microglial cell line (CHME)-3 (**A**) and retinal pigment epithelial cell line (ARPE)-19 (**B**) cells were challenged with both multidrug-resistant *Pseudomonas aeruginosa* (MDR-*PA*) and sensitive *Pseudomonas aeruginosa* (S-*PA*) at an MOI of 10:1, and at indicated time points the supernatant was collected and cells were lysed. Bacterial load and viability were enumerated by plate count method. Student’s *t*-test was used for the analysis, and values are represented as the mean colony-forming units (CFU ± SD) from three sets of independent experiments with *P. aeruginosa* (MDR-*PA* and S-*PA*) strains in CHME-3 and ARPE-19 cells at various time intervals. ** *p* < 0.01, * *p* < 0.05.

Since microglia and RPE cells have the ability to phagocytose bacteria, we assessed the bacterial viability and intracellular proliferation of MDR-*PA* and S-*PA* strains in both cell lines. The enumeration of colony-forming units (CFUs) at various times post-infection (2–24 h) revealed a 1–3 log increase in bacterial burden in cells challenged with MDR-*PA* versus S-*PA* (Figure 1A,B), specifically at the later time points (12 h and 24 h), with the intracellular bacterial burden being higher in cells challenged with MDR-*PA* vs. S-*PA* after inoculation (10.65 log CFU/mL vs. 8.66 log CFU/mL; *p* = 0.008, 12 h; 10.60 log CFU/mL vs. 8.99 log CFU/mL; *p* = 0.01, 24 h in microglial cells, and 10.99 log CFU/mL vs. 8.95 log CFU/mL; *p* = 0.009, 12 h and 11.23 log CFU/mL vs. 9.66 log CFU/mL.; *p* = 0.01, 24 h in RPE cells). Collectively, these data show that in comparison to drug-sensitive strains, resistant bacterial strains grow more in infected cells, indicating increased fitness of multidrug-resistant strains.

### 3.2. Pseudomonas Infection Resulted in Induced Expression of TLRs in Microglia

**Figure 2:**
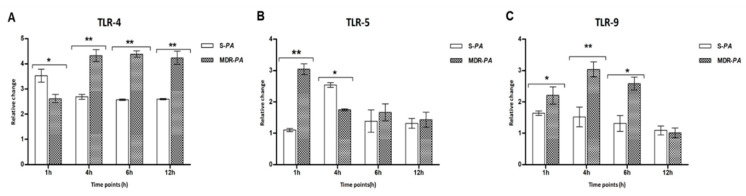
CHME-3 cells were infected with S-*PA* and MDR-*PA*, with an MOI of 10:1. At the indicated time points, the cells were collected and processed for RNA isolation and cDNA synthesis, followed by RT-qPCR. RT-qPCR results shows the differential expression of Toll-like receptor (TLR)4 (**A**), TLR5 (**B**), and TLR9 (**C**) by human microglia infected with clinical strains of MDR-*PA* and S-*PA* at different time points. The data was shown as the mean ± SE from three sets of independent experiments. ** *p* < 0.01, * *p* < 0.05.

Immune cells, including RPE and microglia, are known to express TLRs (Figure S1), which play a central role in recognizing pathogen-associated molecular patterns (PAMPS). Thus, we assessed the modulation of TLRs in CHME-3 cells infected with resistant or sensitive strains. Among various TLRs, we observed that cells challenged with MDR-*PA* exhibited higher levels of TLR4, TLR5, and TLR9, compared to S-*PA* (Figure 2). However, no significant difference was observed in the mRNA levels of TLR1, TLR3, TLR6, or TLR7. Interestingly, the expression of TLR5 peaked as early as 1 h (>3-fold; *p* = 0.004) in cells challenged with MDR-*PA*, followed by gradual decline, whereas S-*PA* showed its highest expression at the 4 h time point.

### 3.3. Differential Expression of Inflammatory Mediators in Microglia Challenged with MDR-PA or S-PA

**Figure 3:**
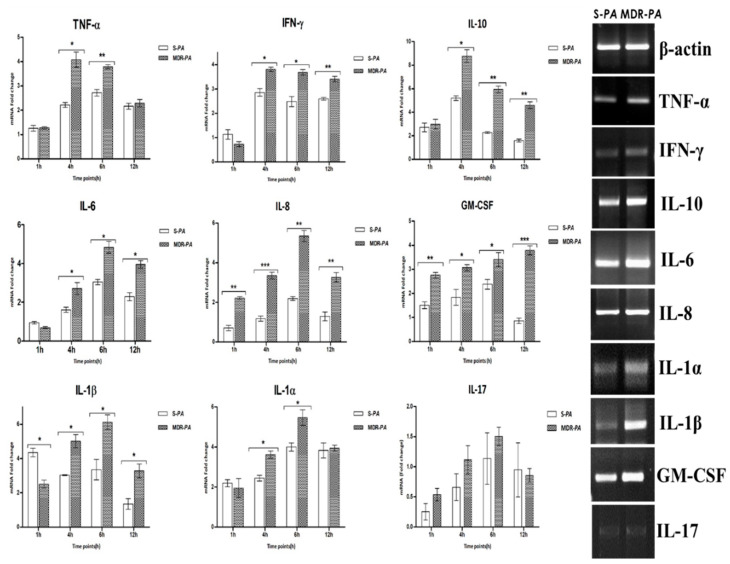
CHME-3 cells were infected with S-*PA* and MDR-*PA*, with an MOI of 10:1. At indicated time points, the cells were collected and processed for RNA isolation and cDNA synthesis, followed by RT-qPCR. Human microglia were infected with clinical MDR-*PA* and S-*PA* strains. The MDR-*PA* strain exhibited persistently higher inflammatory mediators compared to S-*PA*. Student’s *t*-test was used for statistical analysis. The data was shown as the mean ± SE from three sets of independent experiments. *** *p* < 0.001, ** *p* < 0.01, * *p* < 0.05.

Similar to TLR expression, the MDR-*PA* strain showed a concomitant increased expression of cytokines IL-6, IL-8, IL-10, IL-1β, IL-1α, M-CSF, TNF-α, and IFN-γ in CHME-3 cells, compared to the S-*PA* strain (Figure 3). No significant difference in expression was observed for IL-17. While IFN-γ was overexpressed in cells infected with MDR-*PA* compared to the S-*PA* strain at 4 h, 6 h, and 12 h post-infection (>3-fold, *p* = 0.01), TNF-α was also significantly expressed in MDR-*PA* infected microglia cells at 4 h (4-fold vs. 2.2, *p* = 0.04), 6 h (3.8-fold vs. 2.7, *p* = 0.006) and 12 h (2.8-fold vs. 2.2, *p* = 0.04) post-infection. At 4 h post-infection, MDR-*PA*-challenged CHME-3 cells showed their highest mRNA expression (8.8-fold vs. 5.2 fold, *p* = 0.01) of IL-10, compared to cells infected with S-*PA*, indicating a suppression or dampening of the immune response, leading to alteration of pro- and anti-inflammatory cytokines. IL-6, IL-8, and GM-CSF also exhibited significant upregulation compared to the sensitive strain all the time points post-infection, as shown in Figure 2. Real-time PCR results show increased mRNA expression of IL-1β by CHME-3 cells infected with both MDR-*PA* and S-*PA* strains, while expression was significantly greater in cells infected with MDR-*PA* at 4, 6, and 12 h post-infection. Interestingly, cells infected with MDR-*PA* also induced significantly higher expression of IL-1α in CHME-3 cells at 4 h and 6 h post-infection.

### 3.4. Cytokine/Chemokine Protein Expression Following Infection

**Figure 4:**
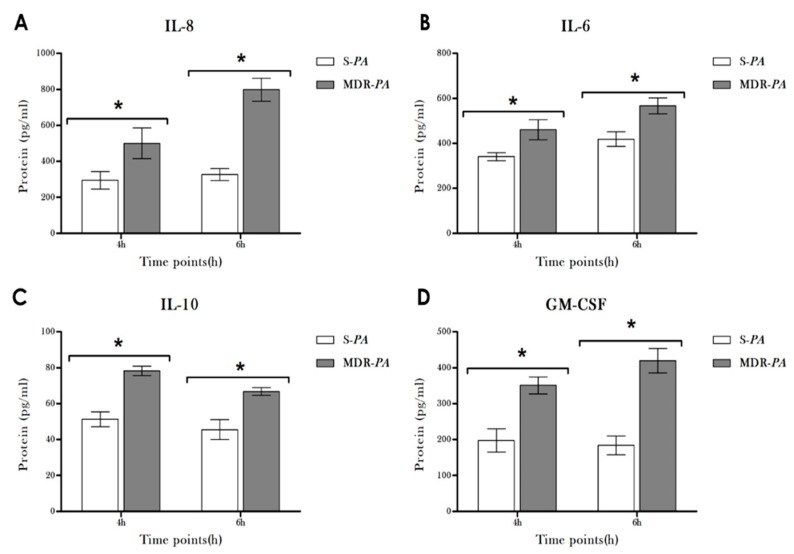
CHME-3 cells were challenged with S-*PA* and MDR-*PA* for the displayed time points. The level of cytokines (**A**) IL-8, (**B**) IL-6, (**C**) IL-10, (**D**) and GM-CSF in the culture media was determined by measuring the concentration using ELISA. Data represent the mean ± SE The values are pooled from three sets of independent experiments. Statistical significance: * *p* < 0.05.

To validate if changes observed at the mRNA level were also observed at the protein level, the concentrations of cytokines and chemokine proteins (GM-CSF, IL-10, IL-6, and IL-8) in supernatants of CHME-3 cells were assessed by ELISA. Consistent with RT-PCR data, significantly increased concentrations of GM-CSF, IL-10, IL-6, and IL-8 accumulated in the culture media of stimulated CHME-3 cells at 4 h and 6 h, respectively (Figure 4). The induction of GM-CSF levels in particular showed elevated levels in cells infected with MDR-*PA* compared to the S-*PA* strain (419.63 ± 33.97 pg/mL vs. 183.82 ± 26.47 pg/mL; *p* = 0.05) at both 4 h and 6 h post-infection.

**Figure 5:**
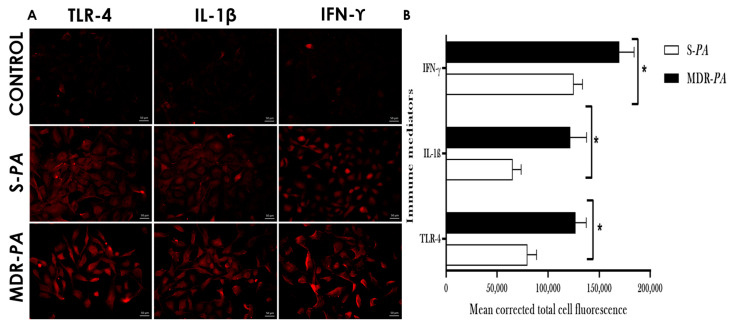
CHME-3 cells were infected with S-*PA* and MDR-*PA* at an MOI of 10:1 and stained with anti-TLR4, -IL-1β, and -IFN-γ antibodies, and analyzed by immunofluorescence microscopy (**A**). Uninfected CHME-3 cells were taken as a control. (**B**) Relative immunofluorescence staining of TLR4, IL-1β, and IFN-γ was quantified from six randomly selected fields. * *p* < 0.05.

Additionally, we assessed the increased expression of TLR-4, IL-1β, and IFN-γ by the MDR-*PA* strain compared to S-*PA* at 4 h post-infection, by immunofluorescence assay, as shown in Figure 5, which correlated well with the mRNA transcription levels. In general, cells infected with MDR-*PA* showed a significantly higher production of mediators as compared with that of S-*PA* strain.

### 3.5. Differential Expression of TLRs and Inflammatory Mediators in Human Retinal Pigment Epithelium (RPE) Cells

**Figure 6:**
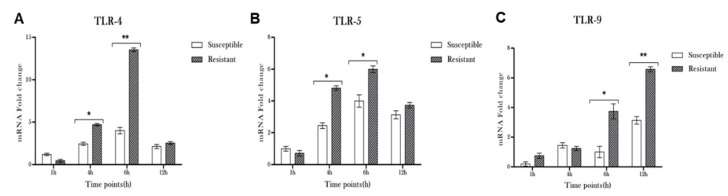
ARPE-19 cells infected with MDR-*PA* and S-*PA* cells were collected at indicated time points, followed by RNA isolation. The RT-qPCR results show the differential expression of TLR4 (**A**), TLR5 (**B**), and TLR9 (**C**). The data are presented as fold change, using a baseline value of 1 for the uninfected cell line. The data is shown as the mean ± SE from three sets of independent experiments; ** *p* < 0.01, * *p* < 0.05.

To check if the differential innate responses observed in microglia cells infected with MDR-*PA* and S-*PA* was a cell-specific response or not, we challenged ARPE-19 cells with the same strains. Similar to CHME-3 cells, we observed that ARPE-19 cells challenged with MDR-*PA* exhibited higher levels of TLR4, TLR5, and TLR9, compared to S-*PA* (Figure 6), and no significant difference was observed in the mRNA levels of TLR2, TLR3, TLR6, TLR7, and TLR8. While the expression of TLR5 showed a gradual increase at 4 h (4.81-fold; *p* = 0.04) and peaked at 6 h (6-fold; *p* = 0.05) in cells challenged with MDR-*PA*, there was relatively low expression at the 4 h and 6 h time points in cells infected with S-*PA*. TLR-4 also similarly showed increased expression in cells challenged with MDR-*PA* (13.53-fold) compared to S-*PA* strain (4-fold) at the 6 h time point. Additionally, TLR9 expression was at its maximum at 6 h and 12 h (6.57-fold; *p* = 0.002) in MDR-*PA*-infected ARPE-19, whereas the S-*PA*-infected cells showed only a 3.13-fold expression, as shown in Figure 6.

**Figure 7:**
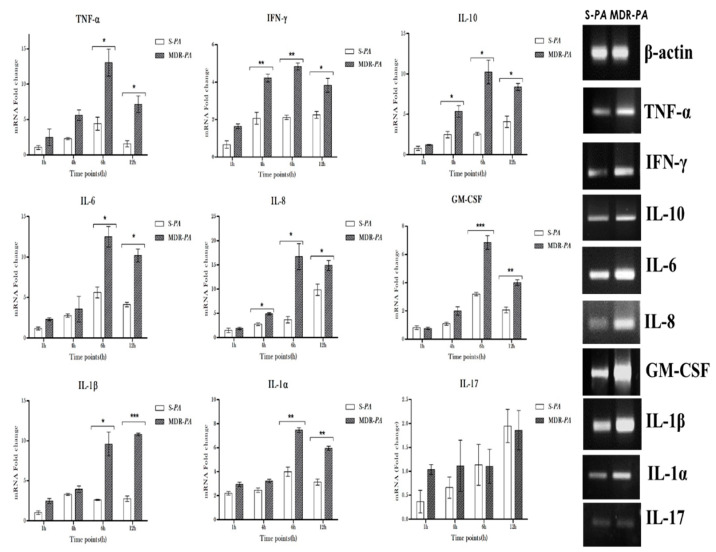
Innate responses of RPE cells infected with S-*PA* and MDR-*PA.* Human Retinal pigment epithelium was challenged with clinical MDR-*PA* and S-*PA* strains. The MDR-*PA* strain exhibit persistently higher inflammatory mediators compared to S-*PA*. Student’s *t*-test was used for statistical analysis. The data is shown as the mean ± SE from three sets of independent experiments and *** *p* < 0.001, ** *p* < 0.01, * *p* < 0.05.

**Figure 8:**
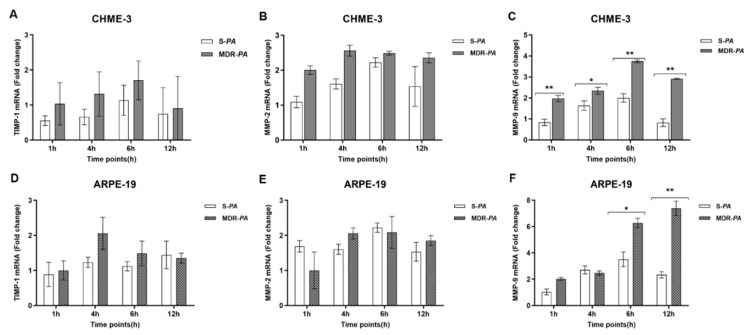
CHME-3 and ARPE-19 cells were challenged with S-*PA* and MDR-*PA*, with a MOI of 10:1. At indicated time points, the cells were collected and processed for RNA isolation; cDNA synthesis followed by RT-qPCR. The bar graphs show that there was no significant expression of MMP-2 (**A**,**D**) or tissue inhibitor of metalloproteinases (TIMP)-1 (**B**,**E**) in either CHME-3 or ARPE-19 cells. (**C**,**F**) Significantly elevated expression of MMP-9 was observed after infection of CHME-3 and ARPE-19 cells with MDR-*PA* and S-*PA* strains. The data are shown as the mean ± SE from three sets of independent experiments; ** *p* < 0.01, * *p* < 0.05.

Moreover, ARPE-19 cells also elicited significantly higher mRNA expression levels of many mediators, as shown in Figure 7. TNF-α was significantly expressed in MDR-*PA*-infected RPE cells at 6 h (13.03-fold vs. 4.41-fold; *p* = 0.02) and 12 h (7.15-fold vs., 1.55-fold; *p* = 0.02) post-infection, while activation of IFN- γ was observed up to 12 h (>3-fold; *p* = 0.01). IL-10 expression was significantly higher in ARPE-19 challenged with the MDR-*PA* at 6 h (10.23-fold vs. 2.57-fold; *p* = 0.03) along with both IL-6 (12-fold; *p* = 0.05) and IL-8 (16.71-fold; *p* = 0.03) at 12 h post-infection, as shown in Figure 8. Again, similar to CHME-3 cells, increased mRNA expression of IL-1β (9.59-fold vs. 2.62-fold; *p* = 0.003) and IL-1α (7.47-fold vs. 4-fold; *p* = 0.008) was also observed by RPE cells infected with MDR-*PA* compared to cells challenged with an S-*PA* strain at a later time point of 6 h, and also compared to microglia cells, where increased expression was observed at 4 h post infection. Additionally, GM-CSF also showed higher mRNA expression in the case of MDR-*PA*, while there was no statistically significant difference in expression for IL-17 (Figure 7).

### 3.6. Differential Gene Expression of Matrix Metalloproteinase-9 in Microglia Cells and RPE Infected with MDR-PA and S-PA Strains

The potential role of matrix metalloproteinases (MMPs) in immunomodulation and inflammation with multidrug resistance strains remains elusive. We observed increased expression of MMP-9, but not TIMP-1 or MMP-2, in both CHME-3 and ARPR-19 cells following infection with *P. aeruginosa* strains (Figure 8). While the levels of MMP-9 expression varied at various time points, MDR-*PA* infected cells showed the maximum expression of MMP-9 at 6 h in CHME-3 cells (3.43-fold vs. 1.1-fold; *p* = 0.005), and at 12 h in ARPE-19 cells (7.39-fold vs. 2.33-fold; *p* = 0.004).

**Figure 9:**
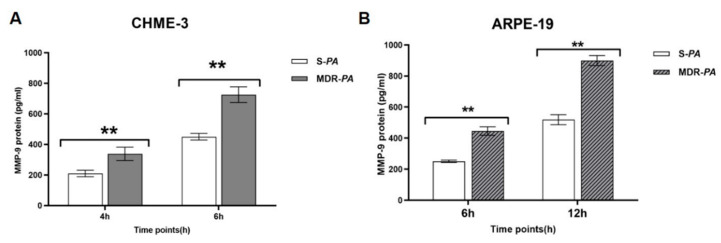
CHME-3 and ARPE-19 cells were challenged with S-*PA* and MDR-*PA*, with an MOI of 10:1. At indicated time points, the cells’ supernatants were collected and processed for ELISA. Significantly elevated MMP-9 levels were observed after infection of (A) CHME-3 and (B) ARPE-19 cells with MDR-*PA* and S-*PA* strains. The data are shown as the mean ± SE from three independent experiments; ** *p* < 0.01, * *p* < 0.05.

The protein expression of MMP-9 in the supernatants of both microglia and RPE cells were also confirmed by ELISA, as shown in Figure 9.

### 3.7. Clustering and Pathway Analysis

**Figure 10:**
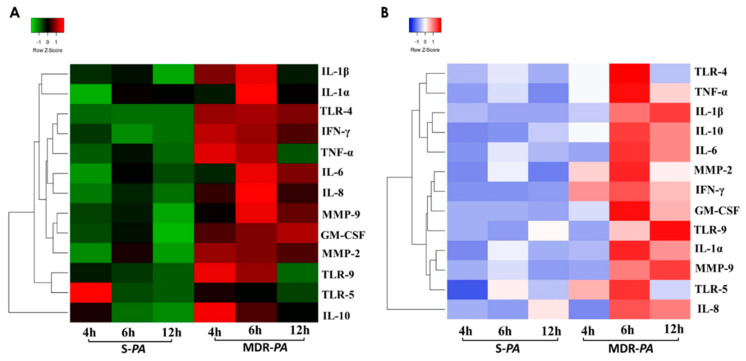
Heatmaps of differential expressions in cytokine/chemokine/growth factor/matrix metalloproteinase (MMP) secretion after S-*PA* and MDR-*PA* infection. (**A**) Human microglia cell line CHME-3 and (**B**) RPE cell line ARPE-19 were infected with S-*PA* and MDR-*PA* for the indicated timepoints. Cells were harvested at 4 h, 6 h, and 12 h, and analyzed. Red denotes increased protein expression levels compared to the geometric mean; green and blue represent decreased protein expression levels compared to the geometric mean. The color intensity correlates with the magnitude of the calculated fold change. Hierarchical cluster analysis of cytokine profiling data is based on Euclidean distance and Ward linkage.

To provide further insight into the changes in the microglial and RPE cells, and to further analyze the differential cytokine expressions induced by the MDR strain of *Pseudomonas* *asaeruginosa*, we performed a hierarchical cluster analysis to draw a heatmap between cytokine responses in both CHME-3 and ARPE-19 cells. Complete linkage and Euclidean distances were employed as metrics to draw association dendrograms between cytokines expressed between MDR-*PA* and S-*PA* strains. Cluster analysis and heatmaps were obtained using Heatmapper (http://www2.heatmapper.ca/). The hierarchical heatmap (Figure 10) shows that there was a clear distinction between the relative cytokine expression of the MDR-*PA* and S-*PA* strains at the time points analyzed. Infection with the S-*PA* strain was found to trigger a comparatively lower induction of inflammatory mediators in the CHME-3 cells compared to infection by the MDR-*PA* strain. Additionally, the highest expression of inflammatory mediators was observed at the 4 h and 6 h time points in MDR-*PA*-infected microglia cells, but comparatively, MDR-*PA*-infected RPE cells showed the highest expression at the 6 h and 12 h time points, as shown in Figure 10.

**Figure 11:**
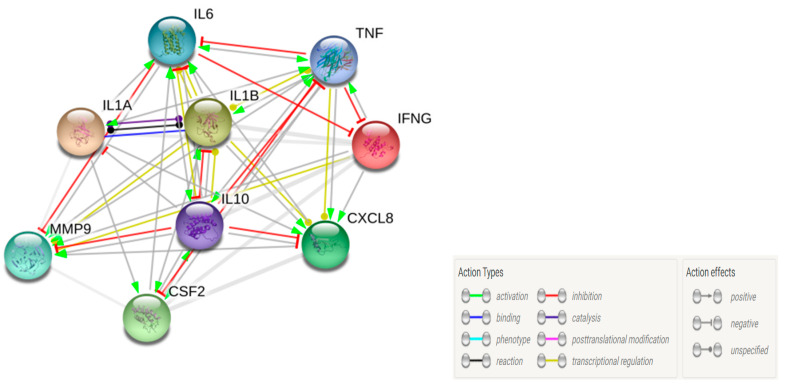
Network analysis of the host immune response in CHME-3 cells and ARPE-19 following *Pseudomonas aeruginosa* infection. Here is an interaction network built using the STRING database (V10.5) of MDR-*PA*-induced changes in inflammatory profiles. The known or predicted three-dimensional (3D) structures of the proteins are displayed inside the circles. The types of interaction evidence are indicated beside the network figure: an arrow line represents positive action, the T-type line represents negative action, and the round headline represents unspecified action. A yellow line represents transcriptional regulation, the red line represents inhibition, the blue line represents binding, and a grey line means reaction.

To further explore the interactions between these selected immune cytokines/chemokines/growth factors/MMPs, STRING (version 10.5; available at https://string-db.org/) analysis was carried out to predict the network, and a close strong interactions were found between the cytokines (Figure 11). With an average local clustering coefficient of 1, and protein–protein interaction (PPI) enrichment *p*-value < 2.25 × 10^−11^, our interactome analysis of MDR-*PA* strain -modulated IL-1β, IL-1α, IL-6, IL-10, IL-8, GM-CSF, MMP-9, TNF-α, and IFN-γ demonstrates that the network formed by these molecules exhibits significantly higher interactions (*n* = 72) between each other (*n* = 9), thus indicating the cytokines are at least partly biologically connected as a group, and are involved in the immune response and cytokine-mediated signaling pathway during infection. Most of these proteins are transcriptional regulators (Figure 11, yellow line), and except for MMP-9 and IL-8, the other immune mediators are involved in the positive regulation of cytokine production, while IL-1α, IL-1β, IL-6, TNF-α, and IL-10 are also involved in the regulation of chemokine production. MMP-9, IL-6, TNF-α, and IL-1β were also predicted to interact with IL-10, an anti-inflammatory cytokine, to play a role in cytokine migration.

## 4. Discussion

The increasing incidence of multidrug-resistant, Gram-negative bacilli characterized by a rapid fulminant course and poor visual and anatomical outcome despite prompt treatment poses a significant challenge for the treatment of endophthalmitis caused by *Pseudomonas aeruginosa* [18]. While the landmark Endophthalmitis Vitrectomy Study, conducted between 1990 and 1994, showed the presence of Gram-negative bacteria in only 4% of the total postoperative endophthalmitis cases [19], two studies from India, including one from our institute [1,20], have revealed a higher proportion of Gram-negative isolates in culture-proven endophthalmitis, with *Pseudomonas* spp. Being the predominant Gram-negative organism in both the studies. It accounts for almost 72.73% of multidrug-resistant (MDR) bacterial endophthalmitis cases [1]. This emergence of MDR *Pseudomonas* strains with high ocular morbidity warrants studies aimed at identifying factors associated with drug resistance and the need for novel treatment strategies. An emerging but often overlooked factor in the process of eradicating pathogenic bacteria from the body is the proper functioning of host innate immunity, along with antibiotic treatment. Most of the research done on MDR bacteria have focused extensively on genetic and epidemiological characterization [21,22,23], and limited studies have actually focused on host–pathogen interactions. This could be attributed to the assumption that the virulence and pathogenicity of MDR and non-MDR bacteria are similar. However, emerging reports now suggest that the pathogenicity and virulence can be remarkably different between MDR and sensitive strains of the same bacterial species [8,24], which raises questions on the role of immune effectors during MDR bacterial infections. A previous study [25] has shown that the presence of an immune response can reduce the requirement of suitable drug concentrations to eliminate the sensitive pathogens, but is insufficient to remove the partially resistant population. Some earlier reports have suggested the killing and binding of bacterial pathogens, including resistant strains, in culture by macrophages, which in turn activates a signaling cascade, resulting in the production of inflammatory cytokines and thereby orchestrating a robust immune response [26,27]. These reports also suggest that the synergy of direct bacterial cell killing and indirect immune cell recruitment enhances the effective clearance of bacterial infections. Thus, strategies designed to augment the host’s innate immune system would be an important adjunct to existing treatment and care.

Previous studies from our lab [28,29,30] and others [31,32,33] have shown that retinal residential cells orchestrate early innate response, and uncontrolled inflammation can cause tissue damage in bacterial endophthalmitis. Indeed, retinal, microgila, muller glia, and RPE have been involved in innate retinal immunity of endophthalmitis. Several studies have discussed the potential role of microglia in RPE inflammasome activation, and it has also been hypothesized that activated microglia migrate rapidly through the RPE, facilitating cell trafficking between the retina and choroid. Additionally, excessive production of chemokines and cytokines from the RPE results in the translocation of microglia and macrophages into the subretinal space [34,35,36]. Currently, a limited understanding exists regarding the interactions between the host and bacterial factors that contribute to the development of endophthalmitis. However, most of these studies have been focused on Gram-positive bacteria, like *Staphylococcus* and *Bacillus* spp. [29,30,31,32,33]. To our knowledge, there are no studies assessing inflammatory response in retinal cells by Gram-negative bacterial infections, such as *Pseudomonas* spp. The factors contributing to the migration of *P. aeruginosa* into the eye, resulting in endophthalmitis, have been suggested to involve the RPE and microglia during the progression of the disease. Thus, steps must be taken to understand the innate immunity–antibiotic relationships, in order to improve treatments, slow the development of resistance, and discover new therapeutic approaches. In this study, we demonstrated that *P. aeruginosa* strains that were isolated from a patient with endophthalmitis evoked an innate response, characterized by the induced expression of TLRs, MMPs, and various inflammatory cytokines and chemokines, depending on their antibiotic profile. Most importantly, we observed that a multidrug-resistant phenotype of the infecting bacteria induced a greater inflammatory response compared to sensitive strains. This also coincides with the increased survival of MDR-*PA* in infected microglia cells. We also further validated if the differential innate responses observed in microglia cells infected with MDR-*PA* and S-*PA* was a cell-specific response or not, and we observed that even in retinal pigment epithelial cells, the response to MDR infectious stimuli was similar; however it was delayed compared to microglia cells. An earlier study [37] correlated the cytokine levels and bacterial clearance during an intraperitoneal infection in mice with MDR and sensitive isolates, along with reference strains of *P. aeruginosa,* and found that MDR strains induced a greater immune response that was able to effectively clear the infective bacteria at 12 h post-infection (p.i.). This suggested the presence of a fitness trade-off that compromises the pathogenic potentials and virulence of MDR isolates [38]. A previous study analyzing different mechanisms of antibiotic resistance that occur in clinical isolates of *P. aeruginosa*, causing severe bloodstream infections, concluded that acquisition of resistance did not lead to decreased fitness [39], and this correlated well with observations of enhanced survival of MDR-*PA* in infected cells that were made in our study too. These data correlate with the results in this study, which show an exacerbated immune response with MDR strains in both microglia and RPE cells. This difference in results could also be attributed to a large array of virulence factors that dampen or reorient both innate and adaptive immune responses in vivo. Our in vitro analysis would also be facilitated by a more detailed, cell-specific, transcriptomic analysis (e.g., RNA-seq), in order to more fully characterize differences in host innate immune transcriptional response to sensitive versus MDR *P. aeruginosa*. Leech et al. [40] have reported that during a MRSA (methicillin-resistant *Staphylococcus aureus*) infection there is a rapid production of IL-10, which is associated with host survival as well as controlling the bacterial dissemination. Our study similarly shows the increased expression of IL-10 in microglia cells at 4 h p.i. and in RPE cells at 6 h p.i., which also correlates with another report by Lamas et al. [41] that demonstrated that T cells producing IL-10 slowed down the clearance of *Salmonella* infection in *S. enterica*, a Gram-negative foodborne pathogenic bacterium, leading to increased mortality. Thus, we have seen how increased efforts in the last few years have made it possible to better understand the role of some immune components in the response against different MDR bacteria. It has been reported that in the activation of the defense mechanisms, the production of TNF-α, along with IL-1β and IL-6, induces a protective inflammatory response for infection control [10,11]. However, it has been suggested that the decrease in the potential of the immune system’s pro-inflammatory reaction in response to a susceptible strain of bacteria is associated with changes in the immunogenic factors, and is not a consequence of the interactions of PAMPs and the pattern recognition receptors in the cells of the immune system, as was previously hypothesized [42]. Additionally, it is also speculated that a decrease in the threshold for the induction of the pro-inflammatory reaction in response to resistant strains of bacteria, as observed in our study, can be considered as the immune system’s compensatory response to its changed fitness. Therefore, the extent of increase in the threshold for cytokine response induction, associated with virulence-associated molecules, may indirectly serve as an immunological marker of antibiotic resistance. Alternatively, proinflammatory cytokines, such as IL-1β and tumor necrosis factor alpha (TNF-α), could upregulate the expression of nitric oxide, which causes damage to bacterial proteins and DNA, leading to extensive proteolysis and tissue damage [43] by the induction of MMP genes [44,45]. Thus, induced expression of MMP-9 by MDR-*PA* could contribute to enhanced pathogenesis of *Pseudomonas* endophthalmitis. Our data also correlates with McClellan et al. [46], who reported a six-fold upregulation in the MMP-9 response to corneal *Pseudomonas aeruginosa* infection. Additionally, Ramsey et al. [47] reported that in urogenital inoculation of *Chlamydia muridarum* in murine models, differences in their susceptibility to chronic chlamydial disease altered the expression of MMP-9. The challenge is to now elucidate if this effect of MMP is beneficial or detrimental during MDR endophthalmitis.

While mounting of a host’s defense is critical in eliminating bacterial infections, an MDR pathogen can cause a severe inflammatory response, which may be counterproductive and result in loss of the eye itself. Thus, multidrug phenotypes of infecting pathogens should be considered, not only to choose appropriate antibiotics, but also for the objective examinations of immune systems and use of anti-inflammatory therapeutic modulators for a more tangible approach.

## 5. Conclusions

Assessing how pathogens initiate and regulate innate immune signaling during endophthalmitis based on their antibiotic susceptibility, and whether this response is excessive and leads to host-induced pathology, may guide future immunomodulatory therapy. Taken together, our study, for the first time, demonstrates differential innate inflammatory responses of ocular cells in response to sensitive and resistant *PA* strains. Therefore, a better understanding of the effect of MDR strains on retinal cells and an immune-therapeutic approach against MDR endophthalmitis offer promising adjunct therapeutic strategies to existing antibiotic therapy for treating infections caused by MDR Gram-negative bacteria.

## Supplementary Materials

The following are available online at https://www.mdpi.com/2076-2607/8/5/735/s1, Figure S1: Expression of TLRs (TLR1–7 and TLR9), following by RT-PCR on cultured human microglia cell line, Table S1: Clinical and demographic details, along with microbiological profile of the strains selected in the study.
