# Supplementary material for: Multidrug-Resistant Pseudomonas aeruginosa Evokes Differential Inflammatory Responses in Human Microglial and Retinal Pigment Epithelial Cells
Source: Microorganisms. 2020 May 14;8(5):735. doi: 10.3390/microorganisms8050735 (PMC7285153; doi:10.3390/microorganisms8050735)
Supplement: Supplementary file 1 [file microorganisms-08-00735-s001.pdf]

Supplementary Materials

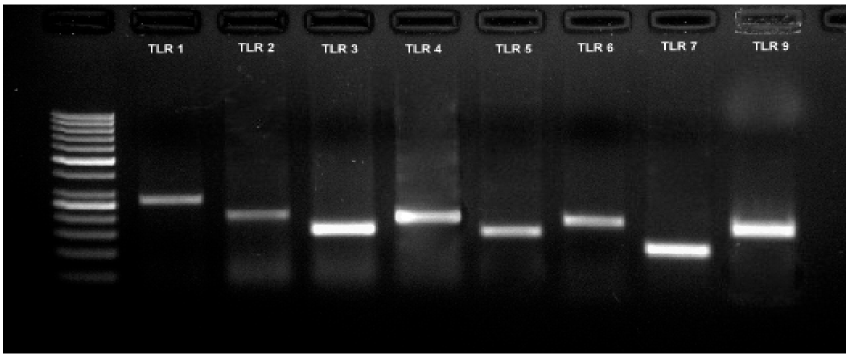

Figure S1. Expression of TLRs(TLR 1-7 and 9), following by RT-PCR on cultured human Microglia cell line.

Table S1. Clinical and Demographic details along with microbiological profile of the strains selected in the study.

Pseudomonas aeruginosa	**Age**	**Gender**	**Diagnosis**	**Microbial Culture**	**Initial Visual acuity**	**Final Visual acuity**	**Antibiotic susceptibility profile with Minimum Inhibitory Concentration (ug/ml)**									**Green Pigmentation**	**β-hemolysis**	**Metallic sheen**
							**CIP**	**GEN**	**OF**	**AK**	**MO**	**GAT**	**CAZ**	**IMP**	**PIT**			
**S-*PA***	63	M	Endogenous	*Pseudomonas aeruginosa*	HM+	CF 1cm	S (≤ 0.25)	S (≤ 1)	S (0.75)	S (≤ 2)	S (≤ 0.25)	S (=2)	S (=3)	S (=2)	S (=8)	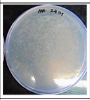	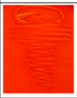	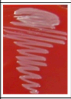
**MDR-*PA***	40	F	Post-operative (Cataract)	*Pseudomonas aeruginosa*	HM+	HM+	R (≥ 4)	R (≥ 16)	R (> 32)	R (≥ 64)	R (= 8)	R (> 32)	R (> 256)	R (=8)	R (≥ 128)	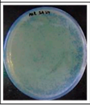	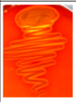	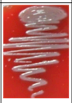

S-*PA*: *Pseudomonas aeruginosa* susceptible strain; MDR-*PA Pseudomonas aeruginosa* resistant strain M: male; F: female, HM+: Hand movement, CF 1cm: Counting finger from 1cm distance, CIP: Ciprofloxacin, GEN: Gentamycin, OF: Ofloxacin, AK: Amikacin, MO: Moxifloxacin, GAT: Gatifloxacin, CAZ: Ceftazidime, IMP: Imipenem, PIT: Piperacillin-tazobactam.
